# Protein intrinsic disorder in plants

**DOI:** 10.3389/fpls.2013.00363

**Published:** 2013-09-12

**Authors:** Florencio Pazos, Natalia Pietrosemoli, Juan A. García-Martín, Roberto Solano

**Affiliations:** ^1^Computational Systems Biology Group, National Centre for Biotechnology, Spanish National Research CouncilMadrid, Spain; ^2^Plant Molecular Genetics Department, National Centre for Biotechnology, Spanish National Research CouncilMadrid, Spain

**Keywords:** protein function, protein structure, protein interactions, protein intrinsic disorder, biological networks, plant environmental responses

## Abstract

To some extent contradicting the classical paradigm of the relationship between protein 3D structure and function, now it is clear that large portions of the proteomes, especially in higher organisms, lack a fixed structure and still perform very important functions. Proteins completely or partially unstructured in their native (functional) form are involved in key cellular processes underlain by complex networks of protein interactions. The intrinsic conformational flexibility of these disordered proteins allows them to bind multiple partners in transient interactions of high specificity and low affinity. In concordance, in plants this type of proteins has been found in processes requiring these complex and versatile interaction networks. These include transcription factor networks, where disordered proteins act as integrators of different signals or link different transcription factor subnetworks due to their ability to interact (in many cases simultaneously) with different partners. Similarly, they also serve as signal integrators in signaling cascades, such as those related to response to external stimuli. Disordered proteins have also been found in plants in many stress-response processes, acting as protein chaperones or protecting other cellular components and structures. In plants, it is especially important to have complex and versatile networks able to quickly and efficiently respond to changing environmental conditions since these organisms cannot escape and have no other choice than adapting to them. Consequently, protein disorder can play an especially important role in plants, providing them with a fast mechanism to obtain complex, interconnected and versatile molecular networks.

## PROTEIN INTRINSIC DISORDER

It is now recognized that a large fraction of the proteome, especially in eukaryotic organisms, lacks a fixed 3D structure in its native form. In these proteins, either the complete chain (“ intrinsically disordered/unstructured protein,” IDP/IUP) or part of it (“ intrinsically disordered/unstructured region,” IDR/IUR) do not adopt a folded structure in its functional form, but exist as a flexible mobile polypeptide (Dunker and Obradovic, 2001; Tompa, 2002; Uversky, 2013).

Intrinsically disordered proteins/intrinsically disordered region were detected from diverse experimental evidences of lack of fixed structure: e.g., missing segments in X-Ray-derived structures or lack of constraints to define a unique structure in NMR. At the sequence level, IDRs are characterized by long stretches of charged and polar residues almost lacking hydrophobic residues, which consequently do not allow the formation of hydrophobic cores to initiate folding (Romero et al., 2001; Dyson and Wright, 2005; Tompa, 2005). This particular (highly biased) amino-acid composition was the basis of the first approaches for detecting IDPs/IDRs from primary sequences. Later, as more examples of experimentally determined IDRs accumulated, specific predictors were trained with them, such as PONDR (Romero et al., 1997) or DISOPRED (Ward et al., 2004). These, together with the latest methodologies based on physical principles, e.g., FoldIndex (Prilusky et al., 2005) and IUPRED (Dosztanyi et al., 2005), constitute the current toolbox for predicting disorder from primary sequences.

Research in this type of proteins was delayed in part by the fact that they apparently contradicted the classic “ structure-function relationship” paradigm, which states that a protein has to be folded in a fixed 3D conformation in order to perform its function. In IDPs, it is actually their lack of structure what is instrumental to perform their particular functions. This is because in most cases the molecular function of these polypeptides is related to transient binding to multiple (different) partners. Such a particular way of interacting could not be achieved by “ fixed” surfaces, but only by those able to adapt to different conformations. Indeed, in many cases IDRs become structured upon binding to a partner, and in some cases the same IDR can adopt different bound structures depending on the partner (Tompa, 2005). This entropy reduction due to the structural gain associated to the binding is in part responsible for the special characteristics of the disorder-mediated interactions. Besides binding, IDRs also act as flexible linkers and “ springs” within the cell (Dunker et al., 2002; Tompa, 2002; Cozzetto and Jones, 2013).

Disordered proteins/regions are associated with key cellular processes such as signaling cascades, transcription regulation, cell cycle control and chaperone activity (Iakoucheva et al., 2002; Uversky et al., 2005; Tompa et al., 2006; Xie et al., 2007). These processes require reversible transient interactions of high specificity and low affinity, eventually with different partners, exactly the type of interactions mediated by these unstructured polypeptides. Consequently, far from being “ rare” or anecdotic, disordered proteins are among the most important proteins in a given proteome, and their mutation is, in many cases, either lethal or leads to diseases (Iakoucheva et al., 2002; Midic et al., 2009). Indeed, the possibility of interacting with multiple partners makes IDPs being “ hubs” (highly connected nodes) in protein interaction networks (Haynes et al., 2006) which are themselves related to lethality (Jeong et al., 2001). For example, the highly studied human transcription factor (TF) p53 is disordered in half of its length and indeed uses these IDRs to interact with its more than hundred different known partners (Oldfield et al., 2008). Similarly, signaling networks are branched and interconnected, and they require transient interactions of high specificity with different partners, making unstructured proteins excellent candidates for them. Another prototypical example are the molecular chaperones, for which a growing body of evidence points to the involvement of disorder in the activity of many of them (Kovacs and Tompa, 2012). Many chaperones contain IDRs (which are involved in the regulation of the chaperone or in the interaction with the substrate itself) or are fully disordered (IDPs). Interacting through disordered segments allows these chaperones to help in the folding of a much broader range of substrates.

Within these long disordered segments, particular stretches of amino-acids, generally with increased evolutionary conservation, have been found to be important for determining the interaction specificity. They can be seen as a sort of “ functional sites” within disordered segments. These include “ molecular recognition features” (MoRFs), which have a tendency to form certain secondary structures (α-MoRFs, β-MoRFs, …) realized when they bind to a partner (Fuxreiter et al., 2004; Mohan et al., 2006), “eukaryotic linear motifs” (ELMs; Gould et al., 2010), and “short linear motifs” (SLiMs; Diella et al., 2008).

These proteins are not only involved in central cellular processes but they are also more abundant than previously anticipated. The development of specific predictors able to detect IDPs/IDRs from primary sequences, and their massive application to complete proteomes rendered surprising results. Almost 1/3 of eukaryotic proteins are mostly disordered and half of them contains at least one long IDR (>30 residues). This rises to 70% for proteins involved in signaling (Iakoucheva et al., 2002; Vucetic et al., 2003; Ward et al., 2004).

Moreover, there is a relationship between disorder content and what one intuitively regards as “ organism complexity.” Even if this is controversial mainly due to the imprecise definition and quantification of “ organismal complexity,” at least there is a clear difference between the relatively low disorder content of prokaryotic organisms and the high disorder found in eukarya (Ward et al., 2004; Schad et al., 2011). This can be related to the involvement of disorder in cellular processes that are apparently more complex and interconnected in higher and multicellular organisms (cell cycle control, signaling cascades, etc.).

Taking together all these observations point toward the involvement of disorder in the generation of the highly-connected and intricate molecular interaction networks which underlie the complex biological processes characteristic of higher organisms. Indeed, protein interactions mediated by IDRs are recognized as a way of introducing plasticity in protein interaction networks (Tompa et al., 2005; Uversky et al., 2005). Along the same line, it has also been shown that in many cases alternative splicing isoforms are characterized by the addition/deletion of IDRs so as to add/remove interacting regions and consequently tune the “ wiring” of the networks these isoforms are involved in (Romero et al., 2006; Buljan et al., 2013).

## PROTEIN DISORDER IN PLANTS

### LARGE-SCALE QUANTIFICATIONS OF DISORDER

In principle, protein disorder in plant proteomes follows the same trends reported for other species. A number of studies focused on plant model organisms showed that disorder is present in the typical processes involving transient interactions with multiple partners. For example, a genome-wide analysis of protein disorder in *Arabidopsis thaliana* (Pietrosemoli et al., 2013) showed that the biological processes more enriched in disordered proteins were related to cell cycle, signaling, DNA metabolism, RNA splicing, etc. In this study, disorder predictions were generated for all proteins in this model organism. These data, together with a functional classification of the proteins in biological processes, allowed to evaluate the degree of disorder of the different biological processes. Carrying out the same process for the Human proteome allows to perform comparative studies on the usage of disorder in both organisms. The proteome of *A. thaliana* follows the expected trend regarding whole disorder content: as an eukaryotic organism, it has much more disorder than bacterial proteomes and, leaving apart discussions on the definition of “ organism complexity” and its quantification, *Arabidopsis* is globally less disordered than *Human*, an organism intuitively regarded as of higher complexity (Schad et al., 2011; Pietrosemoli et al., 2013).

In spite of this lower overall disorder content, there are some biological processes that are more enriched in disorder in *A. thaliana* than in *Human*. Many of these processes are related to the detection and response to external (environmental) stimuli (Pietrosemoli et al., 2013). These include processes related to the perception of light, response to abiotic stress, protein folding (chaperones) and secondary metabolism (mediating plant response to stress). A hypothesis to explain that these processes related to the perception and response to stimuli are more disordered in plants than in organisms of higher complexity involves that plants might have evolved very complex, versatile and intricate systems for interacting with the environment since, being sessile organisms, they cannot escape from environmental hazards and changes, as animals do, and have no other option than responding to them (Pietrosemoli et al., 2013). Protein disorder is a possible way for increasing the “ wiring” (connectivity) of the molecular networks underlying a given biological system. As a consequence, such system becomes more intricate and complex. This relationship between disorder in plants and their increased ability to respond to changing conditions has also been noted by other authors (Sun et al., 2013).

### EXAMPLES OF INVOLVEMENT OF DISORDER IN PLANTS

The involvement of protein intrinsic disorder in a number of plant molecular systems has been studied in detail. Again, in all the cases protein disorder allows the proteins in these systems to interact transiently with multiple partners with high specificity and low affinity. Moreover, in general these systems follow the trend commented above regarding the relationship between disorder in plants and versatility/complexity in the response to stimuli and changing conditions.

Maybe the most studied example of disordered plant systems are the dehydrins (Mouillon et al., 2006; Kovacs et al., 2008; Sun et al., 2013). This large and diverse family of proteins, involved in the response to drought and other environmental stresses, is almost completely disordered: their content of “ standard” secondary structure elements (α-helix and β-strand) is low, and they present a significant content of poly-Pro helices (Mouillon et al., 2006). This family includes protein chaperones such as ERD10 and ERD14 (Kovacs et al., 2008; Tompa and Kovacs, 2010) as well as proteins involved in the binding of metal ions, protection of membranes, and global protection of the cell during the highly compact dry state characteristic of plant seeds (Sun et al., 2013). It looks like dehydrins have evolved for maintaining this disorder state and avoid forming compact folded structures (Mouillon et al., 2006). The conformational flexibility associated to the disordered state allows them to sequester water, ions, proteins (as chaperones), and perform all the other molecular functions associated to their roles in responding to water-related stresses.

Another plant-specific family of proteins heavily relying on disorder for functioning is the GRAS family (Sun et al., 2011; Sun et al., 2012). These proteins play an important role in plant development and are involved in signal transduction cascades, such as those related to hormone response. Within these cascades, they act as integrators of signals (i.e., from different hormones or environmental inputs). It is indeed their disordered region (present in the N-terminal) that allows them to interact with multiple partners through different binding sites (MoRFs, see Introduction) and consequently integrate the signals they represent. The most conserved C-terminal domain of this family (which is actually what characterizes it) is structurally ordered and contains also motifs involved in protein interaction. Among them, there are Leucine-rich regions probably involved in interactions with TFs so as to transduce the integrated signals downstream.

**FIGURE 1:**
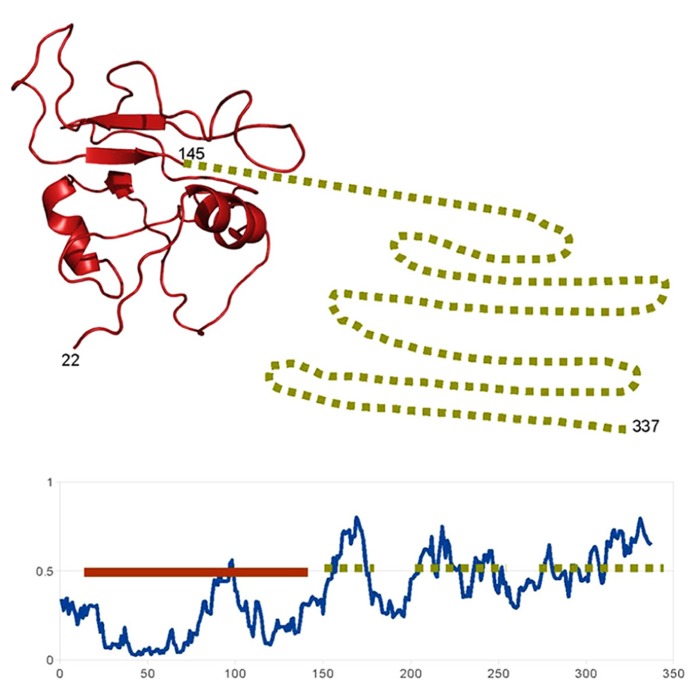
**Example of a highly disordered protein in *A. thaliana*.** Schematic representation of the structural features of the “ putative NAC domain-containing protein 94” (Uniprot: NAC94_ARATH). The disorder prediction of IUPRED (Dosztanyi et al., 2005) show that, according with the standard 0.5 threshold, most of the C-terminal part of the protein (from 150 to 337) is probably unstructured (dotted lines). The N-terminal DNA binding domain is probably structured and, indeed, a structural model can be generated based on the structure of a homolog (PDB:4dul_B, 62% sequence identity; solid line). So probably this protein “ looks like” the representation above: a short structured DNA-binding domain followed by a long flexible disordered region, involved in the binding of different partners.

In some cases, this integration of signals occurs at the level of the TF itself, due to the presence of disordered domains (besides the ordered DNA binding domain), which allows the TF to be influenced by multiple partners. For example the NAC family of plant TF is involved in a variety of processes such as plant defense, stress response or development. These proteins present a conserved (structured) N-terminal DNA-binding domain and a more variable intrinsically disordered C-terminal region (Jensen et al., 2010; Sun et al., 2013; **Figure 1**). This region acquires local structure (α-helix) when binding to the multiple partners of these proteins. This mechanism by which a TF is influenced by multiple partners through disordered regions, which also happens with other plant TFs such as the basic leucine zipper domain (bZIP) family (Yoon et al., 2006), is similar to that of the human p53 commented.

It was also proposed that many chloroplast proteins whose genes were originally encoded by the chloroplast genome acquire disordered regions as they are transferred to the nucleus (Yruela and Contreras-Moreira, 2012). In concordance with its prokaryotic origin, proteins coded in the chloroplast genome almost lack disordered regions. Nevertheless, it looks like the “ eukaryotic machinery” of the nucleus adds disorder to them once they become coded there. This reinforces the idea of the relationship between disorder and the emergence of the complex molecular machineries associated to eukaryotic organisms.

## CONCLUSIONS

In summary, recent research is showing that, in contrast to the classical dogma, intrinsic disorder is an important feature for many proteins to function. In general, protein disorder allows interaction versatility and adds complexity to the interactomes. It is likely a way in which evolution can increase the complexity of biological networks without increasing excessively the size of the genomes. In plants, the predominance of intrinsic disorder in proteins involved in responses to environmental conditions could be explained as a requirement of these processes to be more complex due to the special characteristics of these sessile organisms.

## Conflict of Interest Statement

The authors declare that the research was conducted in the absence of any commercial or financial relationships that could be construed as a potential conflict of interest.
